# Correlation between coronary angioscopy yellow grade and lipid plaque assessment by integrated backscatter intravascular ultrasound

**DOI:** 10.1007/s12928-025-01133-6

**Published:** 2025-05-27

**Authors:** Atsushi Tanita, Shinichiro Sunamura, Tsuyoshi Ogata, Kazuki Noda, Toru Takii, Yoshio Nitta, Seijiro Yoshida, Shigeto Namiuchi

**Affiliations:** 1 Department of Cardiology, Sendai City Medical Center, Sendai Open Hospital, 5-22-1 Tsurugaya, Miyagino-ku, Sendai-shi, Miyagi-ken 983-0824 Japan; 2 Department of Cardiovascular Surgery, Sendai City Medical Center, Sendai, Japan

**Keywords:** Coronary angioscopy, Coronary artery disease, IB-IVUS, PCI

## Abstract

Coronary angioscopy (CAS) enables direct qualitative assessment of the coronary artery lumen, while integrated backscatter intravascular ultrasound (IB-IVUS) provides a quantitative evaluation of coronary plaque tissue characteristics. Despite the utility of both techniques in assessing coronary plaque status, data on the correlation between their findings remain limited. To investigate the association between CAS-derived findings and results obtained through IB-IVUS. This retrospective analysis included 36 patients who underwent both CAS and IB-IVUS during percutaneous coronary intervention (PCI) at our institution. CAS and IB-IVUS were performed on the same coronary artery treated during PCI. Plaques were categorized into four groups based on their yellow color grade using CAS. For the IB-IVUS analysis, measurements were performed at the minimum lumen diameter site of the culprit lesion. A significant correlation was observed between plaque yellowishness and plaque characteristics on IB-IVUS. Higher plaque yellowishness was associated with an increased percentage of all lipid pool (P < 0.01), a greater proportion of attenuated plaque (P < 0.01), and a larger estimated lipid plaque volume (P < 0.01). Additionally, plaques with higher yellowishness grades had significantly thinner fibrous caps (P < 0.01). The findings suggest that higher plaque yellowishness observed via CAS correlates with a larger lipid plaque volume and thinner fibrous caps, as assessed through IB-IVUS.

**Graphical abstract:**

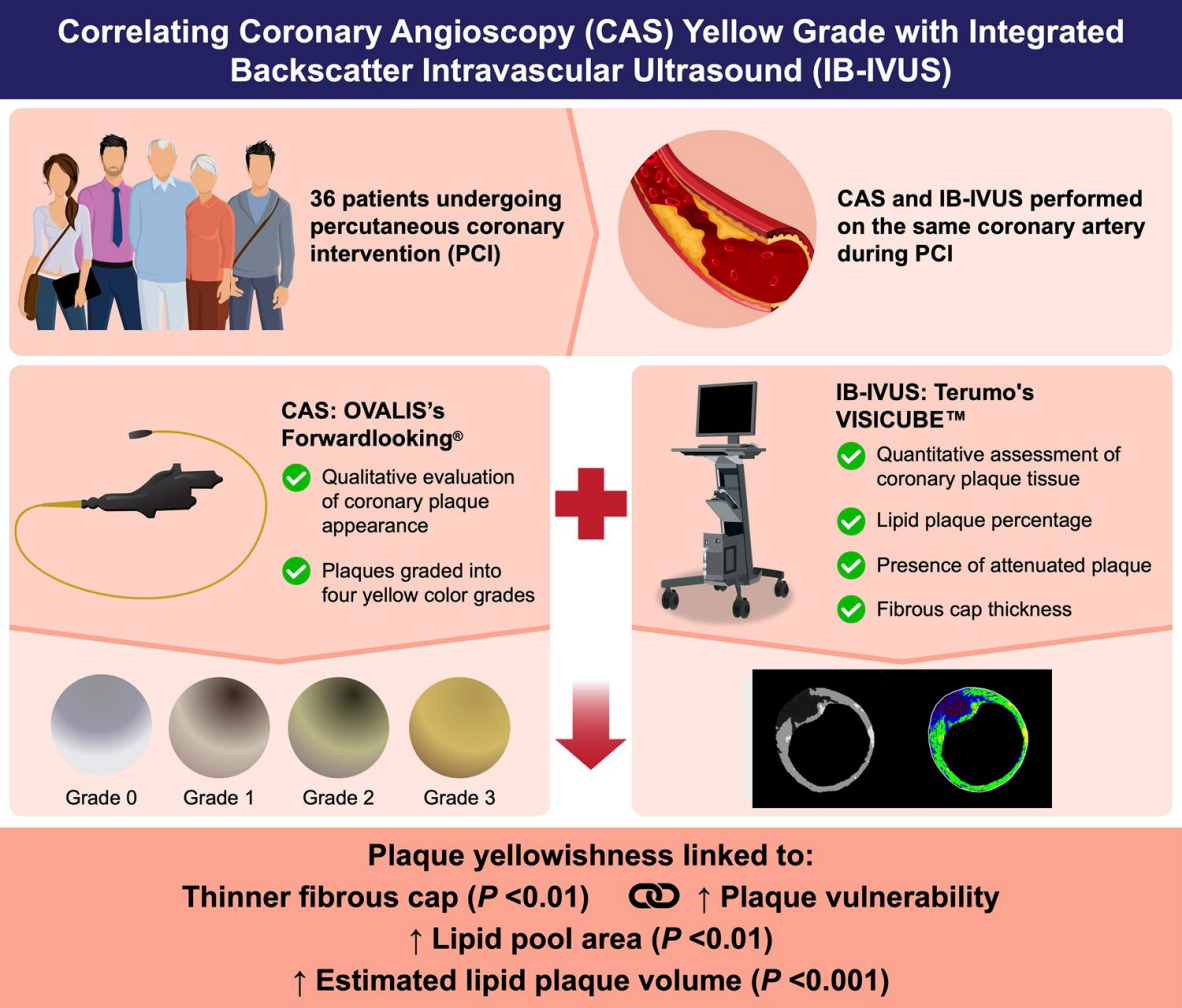

## Introduction

At our institution, coronary angioscopy (CAS) and integrated backscatter intravascular ultrasound (IB-IVUS) were performed simultaneously during percutaneous coronary intervention (PCI) as part of clinical practice for comprehensive lesion assessment, aiming to guide the optimal procedural strategy. CAS enables direct qualitative assessment of the coronary artery lumen. Through CAS, clinicians can visually evaluate the yellowishness of lipid plaques in the coronary arteries, and plaques exhibiting greater yellowishness are considered more vulnerable [1]. In contrast, IB-IVUS calculates the energy of backscattered ultrasound waves reflected from target structures, allowing for quantitative evaluation of coronary plaque tissue characteristics. Previous studies have demonstrated that combining conventional grayscale IVUS with CAS enhances the ability to identify lesion morphology and plaque components [2]. Compared with grayscale IVUS, IB-IVUS provides superior identification of fibrous components and lipid pools within coronary plaques [3]. Although both CAS and IB-IVUS are essential modalities for understanding the pathophysiology of coronary artery disease, limited evidence exists regarding the correlation between their findings. Furthermore, a study using optical coherence tomography (OCT) reported an inverse correlation between the fibrous cap thickness of coronary plaques and the degree of plaque yellow coloration observed by CAS; however, the study did not include data on plaque volume [4]. A comprehensive qualitative and quantitative evaluation of intracoronary plaques enhances the optimization of procedural strategies during PCI, thereby minimizing the risk of complications and improving long-term outcomes. Moreover, advancements in these evaluation techniques are anticipated to enable more precise and personalized management of coronary artery disease. Previous studies, primarily conducted using autopsy specimens, have provided valuable insights. However, no in vivo investigations have explored the relationship between plaque yellowishness, as evaluated by CAS, and the lipid pool size or cap thickness, as assessed by IB-IVUS. In the present study, we aimed to quantitatively evaluate the in vivo association between the degree of yellowishness observed with CAS and the lipid pool size and cap thickness measured using IB-IVUS.

## Methods

### Study population

**Fig. 1:**
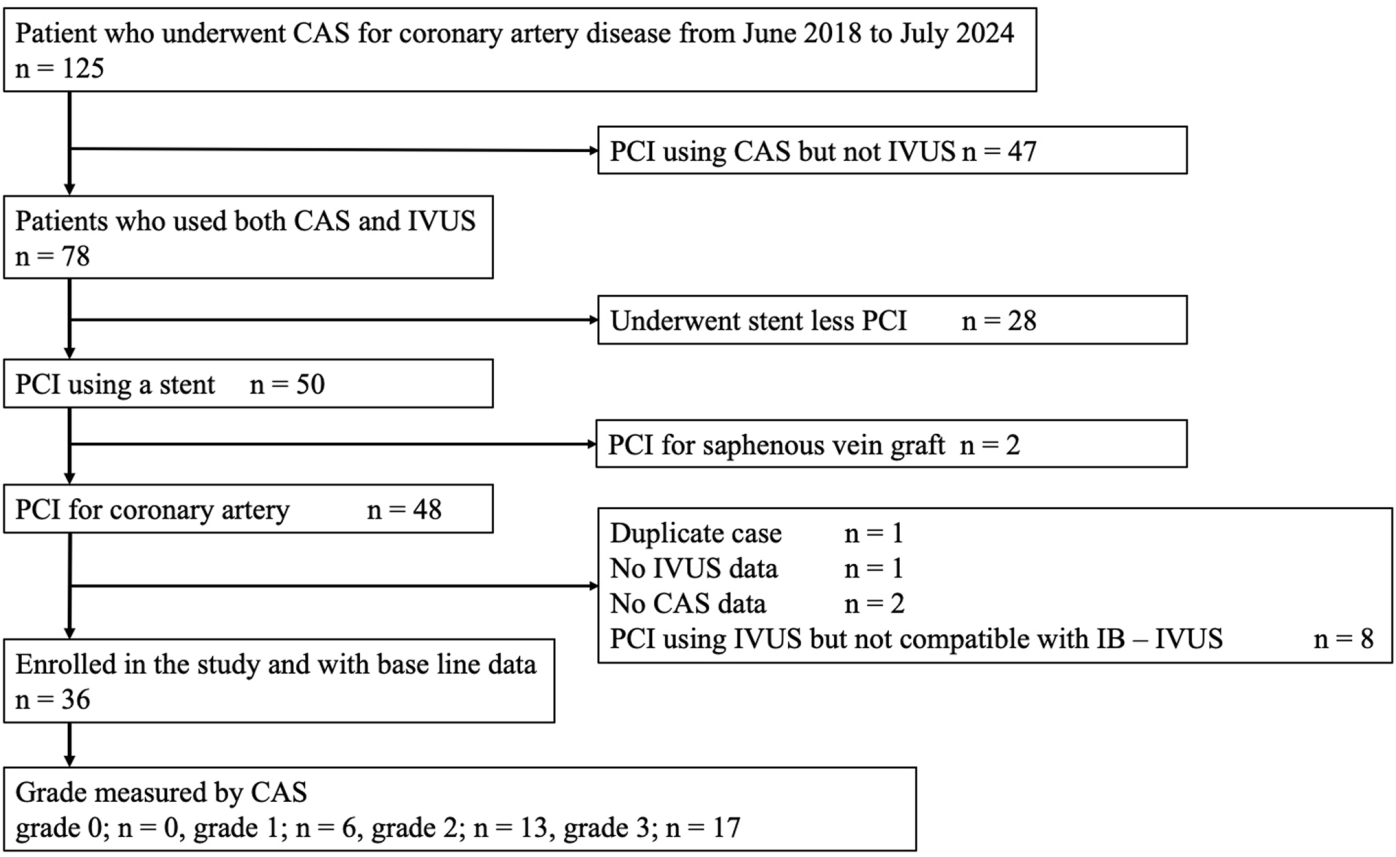
Flow diagram of the study participants. This figure provides a visual summary of participant inclusion and exclusion criteria, detailing the steps from initial enrolment to the final analyzed cohort

This was a retrospective observational study. We conducted a retrospective review of cases in which CAS was performed during PCI at Sendai City Medical Center, Sendai Open Hospital, from June 2018 to July 2024. Cases were excluded if they met any of the following criteria: (1) CAS was performed in combination with OCT instead of IVUS; (2) CAS was the sole imaging modality used during PCI; (3) the procedure involved stent-less PCI; (4) PCI was performed on a saphenous vein graft; (5) a second PCI was performed during the observation period (in such cases, data from the first PCI were included, and the second procedure was excluded); (6) IVUS data were unavailable; (7) CAS data were unavailable; or (8) an IVUS device incompatible with IB-IVUS was used. Figure 1 provides a flow diagram of the study participants.

### Protocol

We analyzed various patient characteristics, including age, sex, body mass index, admission status, and treatment following hospitalization. Diagnoses of hypertension, diabetes mellitus, and dyslipidemia were obtained from medical records or documented patient histories of prior medical therapy. Patients were categorized based on the results of CAS. This investigation adhered to the principles outlined in the Declaration of Helsinki. The study was approved by the ethical committee of Sendai City Medical Center (approval number: 2025-0007), and informed consent was obtained from all patients in accordance with the hospital’s ethical committee guidelines.

### Measurement of CAS grade

In accordance with a previously reported method, [5] the degree of yellowishness of coronary artery plaques was visually evaluated using CAS and graded on a four-point scale (0: white, 1: light yellow, 2: yellow, 3: dark yellow). The testing equipment consisted of the Forward-looking catheter (OVALIS, Osaka, Japan) and the OVALIS CAS imaging system (OVALIS). For coronary imaging, the angioscope was placed in the coronary artery alongside the guidewire, and a 10% low molecular dextran injection was used to clear blood from the lumen. The imaging and grading of plaque yellowishness were conducted by a board-certified cardiologist specialising in catheter interventions.

### Measurement of coronary intervention site by IVUS and color scale of Integrated backscatter IVUS

**Fig. 2:**
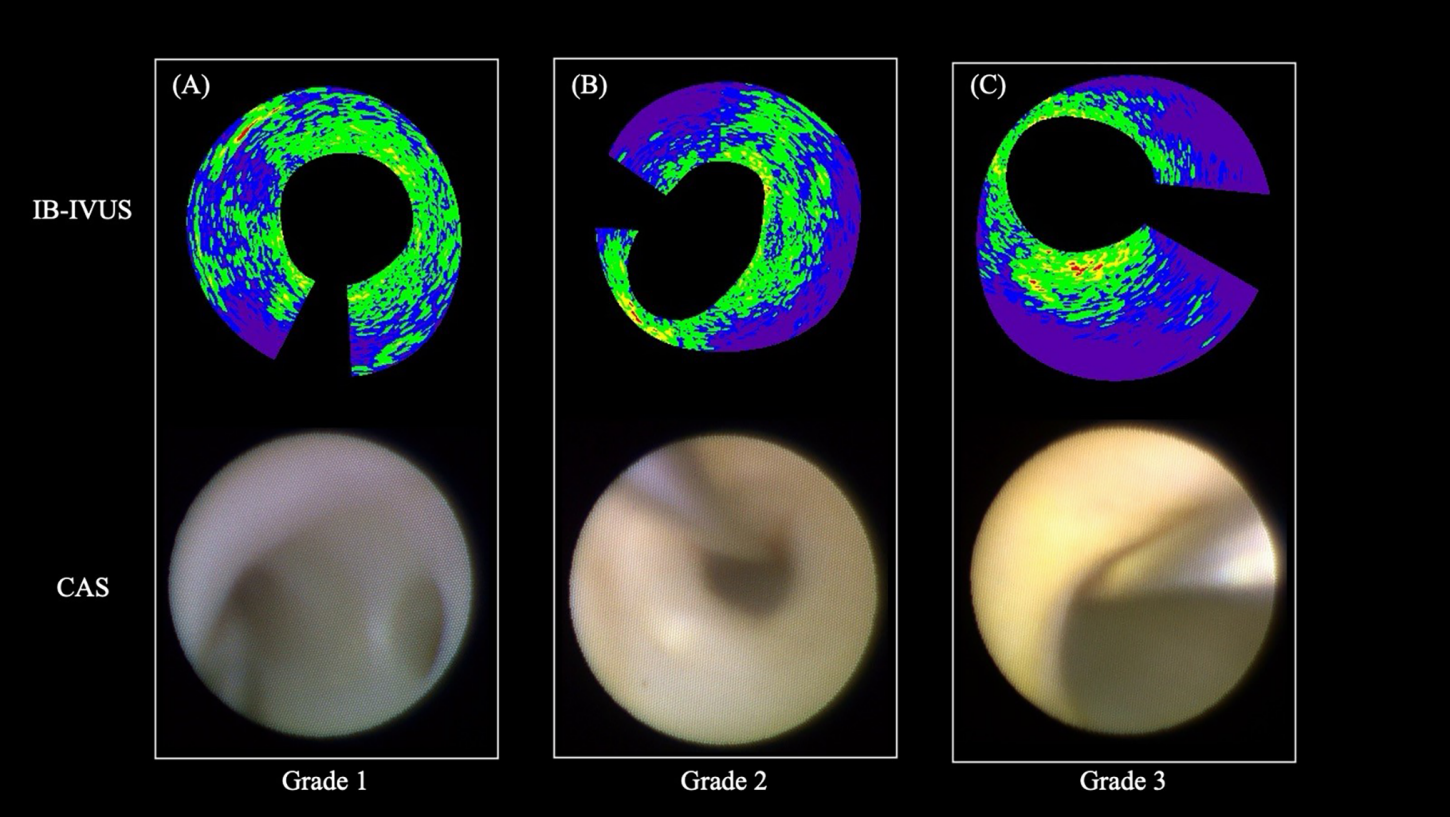
Representative images illustrating the correlation between coronary angioscopy (CAS) and integrated backscatter intravascular ultrasound. **A** CAS image demonstrating a plaque classified as grade 1 (light yellow), **B** CAS image demonstrating a plaque classified as grade 2 (yellow), and **C** CAS image demonstrating a plaque classified as grade 3 (bright yellow)

We used an IVUS and IB-IVUS system [60 MHz (H-mode), VISICUBE, Terumo Corp., Tokyo, Japan] along with a mechanical rotating IVUS catheter (AltaView, Terumo Corp.) to measure and assess lesion-specific target sites within the coronary arteries and their tissue characteristics. In accordance with previous studies, IB values were defined as follows: lipid pool (attenuated), IB <  − 60 decibels; lipid pool, − 60 < IB ≤  − 51 decibels; fibrosis, − 51 < IB ≤  − 38 decibels; dense fibrosis, − 38 < IB ≤  − 31 decibels; and calcification, − 31 decibels ≤ IB. Each tissue type was assigned a unique color—purple for lipid pool (attenuated), blue for lipid pool, green for fibrosis, yellow for dense fibrosis, and red for calcification [6]. Measurements for both IVUS and IB-IVUS were performed at the minimum lumen diameter (MLD) site of the lesion treated with PCI. Using IVUS, we manually traced the external elastic membrane and internal lumen to measure the MLD, minimum vessel diameter, lumen area, vessel area, and plaque area. With IB-IVUS, the proportions of calcification, dense fibrosis, fibrosis, lipid pool, and lipid pool (attenuated) at the MLD site of the target lesion were automatically calculated. The sum of the lipid pool and lipid pool (attenuated) was defined as the ‘all lipid pool’. Additionally, the cap thickness of the lipid plaque was measured by identifying the thinnest fibrous cap within the cross-sectional image at the MLD site. All IVUS and IB-IVUS evaluations were conducted by a board-certified cardiologist specialising in catheter interventions. A representative case demonstrating the correlation between CAS and IB-IVUS is shown in Fig. 2.

### Calculation of estimated lipid plaque volume

Using the IB-IVUS results, the estimated lipid volume was calculated. As defined in a previous study, the ‘estimated lipid volume’ was determined as ‘the lipid area at the MLD site × the total stent length’. In that study, the lipid area in each slice was measured by IB-IVUS using color-coded images, and the lipid volume was calculated by integrating the lipid area of each slice at 1-mm intervals throughout the target lesion, demonstrating a strong correlation with the measured lipid volume [7]. This evaluation method was adopted to calculate the estimated lipid volume in the present study. Accordingly, the estimated lipid volume was calculated by measuring the lipid area at the MLD cross-section of the lesion treated with PCI using IB-IVUS and multiplying it by the length of the stent placed at the same site.

### Evaluation of the correlation between CAS and IB-IVUS

As this was a retrospective study, precisely matching the locations assessed by CAS and IB-IVUS was challenging due to the inherent limitations of angioscopy. For CAS, we focused on the plaque exhibiting the highest degree of yellowish discoloration within the same coronary artery evaluated by IB-IVUS. Although this plaque was not always located exactly at the MLD site, we carefully selected plaques located as close as possible to the stenotic lesion (MLD site). Thus, CAS evaluations were performed on plaques presumed to be immediately adjacent to or near the stenotic lesion, considering the practical limitations of angioscopic examination.

### Statistical analysis

Continuous data are presented as means ± standard deviations or as medians and interquartile ranges, and groups were compared using the Kruskal–Wallis test. Categorical data are presented as percentages, and groups were compared using Fisher’s exact test. A P-value of < 0.05 was considered statistically significant. All statistical analyses were performed using EZR version 1.65 (Saitama Medical Center, Jichi Medical University, Saitama, Japan), a graphical user interface for R version 4.3.1 (The R Foundation for Statistical Computing, Vienna, Austria). EZR is a modified version of R commander designed to add statistical functions frequently used in biostatistics [8]. Missing covariates included left ventricular ejection fraction (n = 1, 2.8%) and HbA1c (n = 1, 2.8%).

## Results

During the study period, 125 patients underwent CAS during PCI, of whom 89 were excluded for the following reasons: PCI using CAS but not IVUS (n = 47), stent-less PCI (n = 28), PCI for a saphenous vein graft (n = 2), duplicate case data (n = 1), missing IVUS data (n = 1), missing CAS data (n = 2), and PCI using IVUS that was incompatible with IB-IVUS (n = 8). Therefore, 36 patients were included in the analysis. The clinical characteristics of these patients are presented in Table 1. Patients were initially categorized into four groups based on CAS findings. However, due to the absence of patients in the CAS grade 0 group, the final analysis comprised three groups: CAS grade 1 (n = 6), CAS grade 2 (n = 13), and CAS grade 3 (n = 17). Additionally, since the clinical characteristics of CAS grades 1 and 2 were similar, we combined these groups for comparison with the grade 3 group. The clinical characteristics of both groups are also summarized in Table 1.

**Table 1:** Baseline characteristics of the patients included in this study and patient characteristics grouped according to the CAS grades

Valuables	Median [IQR]	CAS grade			P-value (1 vs. 2 vs. 3)	CAS grade	P-value (1 + 2 vs. 3)
		1 (n = 6)	2 (n = 13)	3 (n = 17)		1 + 2 (n = 19)	
Age (years)	70 [55−78.3]	70 [67–75]	70 [50–75]	70 [56–79]	0.89*	70 [53–76]	0.96*
BMI (kg/m^2^)	24.9 [23.7−27.0]	23.5 [22.1–24.4]	25.8 [24.4–27.5]	24.8 [23.4–27.0]	0.10*	25.0 [23.8–26.6]	0.79*
Male sex (%)	–	4 (67)	12 (92)	15 (88)	0.29**	16 (84)	1.00**
ACS (%)	–	1 (17)	8 (62)	5 (29)	0.20**	9 (47)	0.53**
Dyslipidemia (%)	–	5 (83)	6 (46)	7 (41)	0.26**	11 (58)	0.51**
Diabetes mellitus (%)	–	1 (17)	3 (23)	3 (18)	1.0**	4 (21)	1.00**
Hypertension (%)	–	5 (83)	9 (69)	7 (41)	0.15**	14 (74)	0.09**
Smoking (%)	–	2 (33)	6 (46)	7 (41)	1.00**	8 (42)	1.00*
LVEF (%)	61 [55–69]	68 [66–69]	61 [54–69]	51 [55–68]	0.71*	65 [56–69]	0.87*
BS (mg/dL)	146 [107–172]	146 [115–152]	171 [128–236]	128 [98–152]	0.08*	126 [59–153]	0.06*
HbA1c (%)	6.0 [5.9–6.6]	6.1 [5.9–6.2]	6.2 [6.0–7.8]	6.0 [5.8–6.5]	0.56*	6.1 [5.9–6.6]	0.44*
TC (mg/dL)	187 [153–232]	144 [141–159]	232 [165–276]	190 [170–222]	0.10*	173 [144–258]	0.98*
HDLC (mg/dL)	44 [38–49]	52 [47–52]	41 [39–49]	41 [36–45]	0.09*	46 [39–51]	0.18*
LDLC (mg/dL)	118 [85–163]	68 [65–92]	159 [108–185]	119 [106–154]	0.12*	115 [68–178]	0.76*
TG (mg/dL)	124 [91–174]	95 [73–116]	129 [91–178]	140 [113–178]	0.15*	121 [79–156]	0.19*
MLD (mm)	1.9 [1.6–2.2]	2.0 [1.6–2.1]	1.7 [1.4–2.4]	1.9 [1.7–2.2]	0.71*	1.7 [1.6–2.2]	0.42*
MVD (mm)	3.9 [3.4–4.4]	3.5 [3.4–3.7]	3.6 [3.1–4.5]	4.1 [3.5–4.5]	0.09*	3.5 [3.2–4.0]	0.04*
Lumen area (mm^2^)	2.9 [2.3–3.8]	3.5 [2.6–3.7]	2.5 [2.3–3.3]	3.1 [2.5–4.0]	0.51*	2.6 [2.3–3.7]	0.69*
Stent length (mm)	24.0 [19.8–32.3]	25.5 [22.0–32.0]	22.0 [19.0–29.0]	28.0 [20.0–38.0]	0.59*	22.0 [20–31]	0.53*
Stent diameter (mm)	3.5 [3.0–3.5]	2.9 [2.6–3.0]	3.0 [2.8–3.0]	3.5 [3.5–3.5]	< 0.01	3.0 [2.8–3.3]	< 0.01*
Vessel area (mm^2^)	12.2 [9.9–15.8]	10.7 [7.8–11.6]	8.8 [8.2–13.4]	15.4 [12.6–18.9]	< 0.01*	10.2 [7.8–12.6]	< 0.01*
Plaque area (mm^2^)	9.5 [6.1–12.0]	6.2 [5.0–8.7]	6.1 [5.5–9.5]	11.9 [10.1–16.6]	< 0.01*	6.1 [5.3–9.5]	< 0.01*
Calcification (%)	0.3 [0.0–0.9]	0.8 [0.5–1.1]	0.5 [0.2–0.9]	0.1 [0.0–0.2]	0.04*	0.6 [0.3–1.1]	0.02*
Dense fibrosis (%)	2.7 [0.7–4.1]	3.4 [2.7–6.2]	4.0 [2.9–4.7]	0.9 [0.4–2.6]	< 0.01*	3.9 [2.7–5.0]	< 0.01*
Fibrosis (%)	30.7 [20.2–37.7]	45.4 [38.0–54.6]	35.9 [29.9–38.5]	21.1 [12.4–30.6]	< 0.01*	37.5 [30.5–45.4]	< 0.01*
Lipid pool (%)	28.5 [24.7–32.1]	29.0 [27.0–31.4]	25.3 [22.0–31.3]	28.7 [25.7–32.6]	0.76*	28.3 [23.2–31.6]	0.64*
Lipid pool (attenuated) (%)	33.0 [27.5–50.5]	19.7 [10.3–28.1]	31.2 [21.0–34.1]	50.5 [34.7–60.1]	< 0.01*	29.8 [16.4–32.3]	< 0.01*
All lipid pool (%)	65.2 [56.1–78.1]	50.6 [38.2–58.4]	59.4 [49.8–64.2]	76.7 [68.1–87.0]	< 0.01*	56.8 [47.6–2.6]	< 0.01*
Cap thickness (mm)	0.3 [0.2–0.5]	0.6 [0.4–0.6]	0.4 [0.4–0.6]	0.2 [0.1–0.2]	< 0.01*	0.5 [0.4–0.6]	< 0.01*
Lipid pool area (mm^2^)	6.2 [3.9–9.2]	2.7 [2.3–5.0]	4.3 [2.5–6.1]	9.1 [6.7–13.6]	< 0.01*	4.3 [2.4–5.9]	< 0.01*
Estimated lipid volume (mm^3^)	161.1 [83.9–266.0]	69.1 [49.9–183.3]	86.1 [73.9–137.0]	278.6 [178.1–388.7]	< 0.01*	84.4 [56.3–150.6]	< 0.01*

*CAS* coronary angioscopy; *IQR* interquartile range; *BMI* body mass index; *ACS* acute coronary syndrome; *LVEF* left ventricular ejection fraction; *BS* blood sugar; *HbA1c* hemoglobin A1c; *TC* total cholesterol; *HDLC* high-density lipoprotein cholesterol; *LDLC* low-density lipoprotein cholesterol; *TG* triglycerides; *MLD* minimum lumen diameter; *MVD* mean vessel diameter

*Mann–Whitney U test

**Fisher’s exact test

### Comparison of CAS grade and IB-IVUS results

**Fig. 3:**
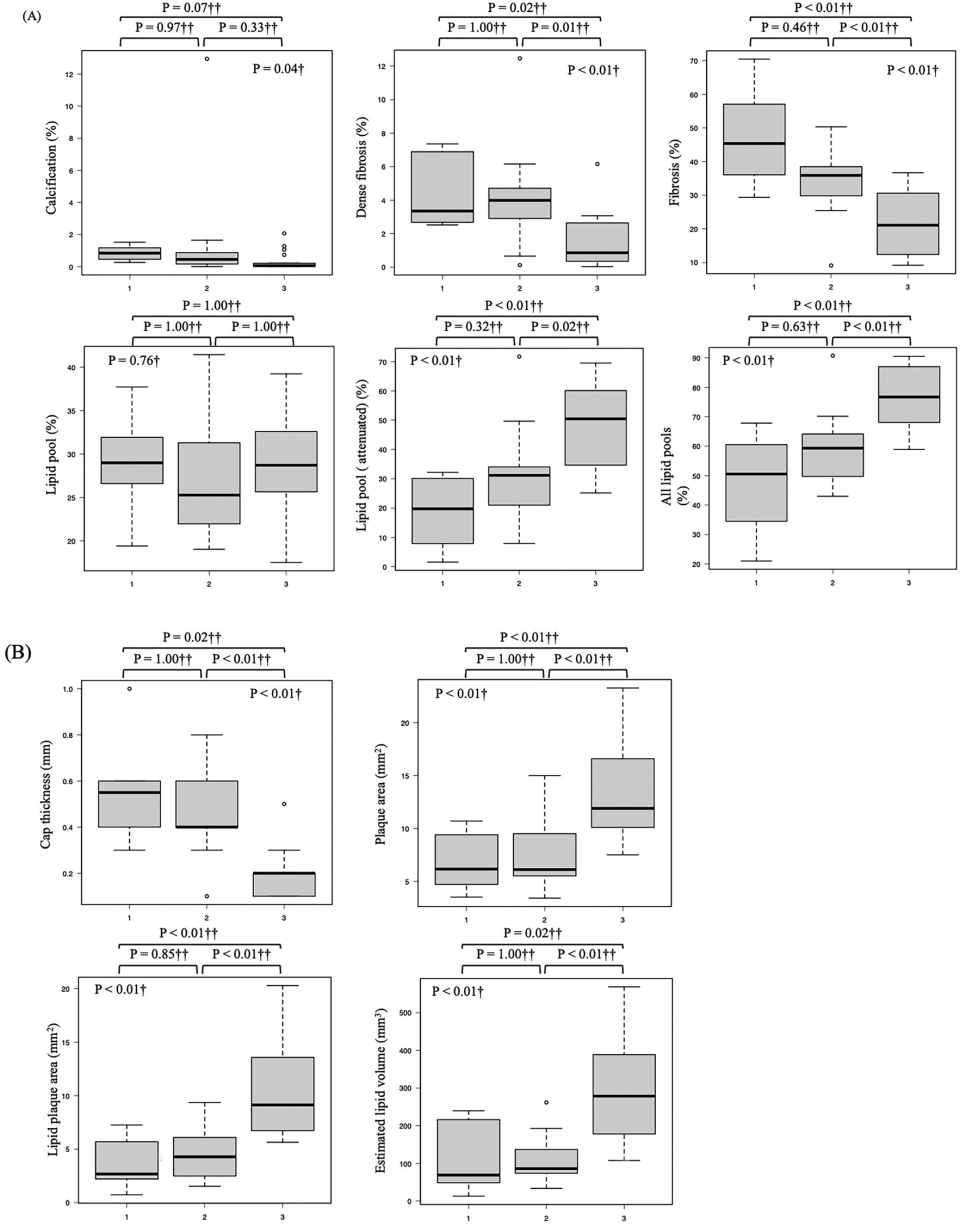

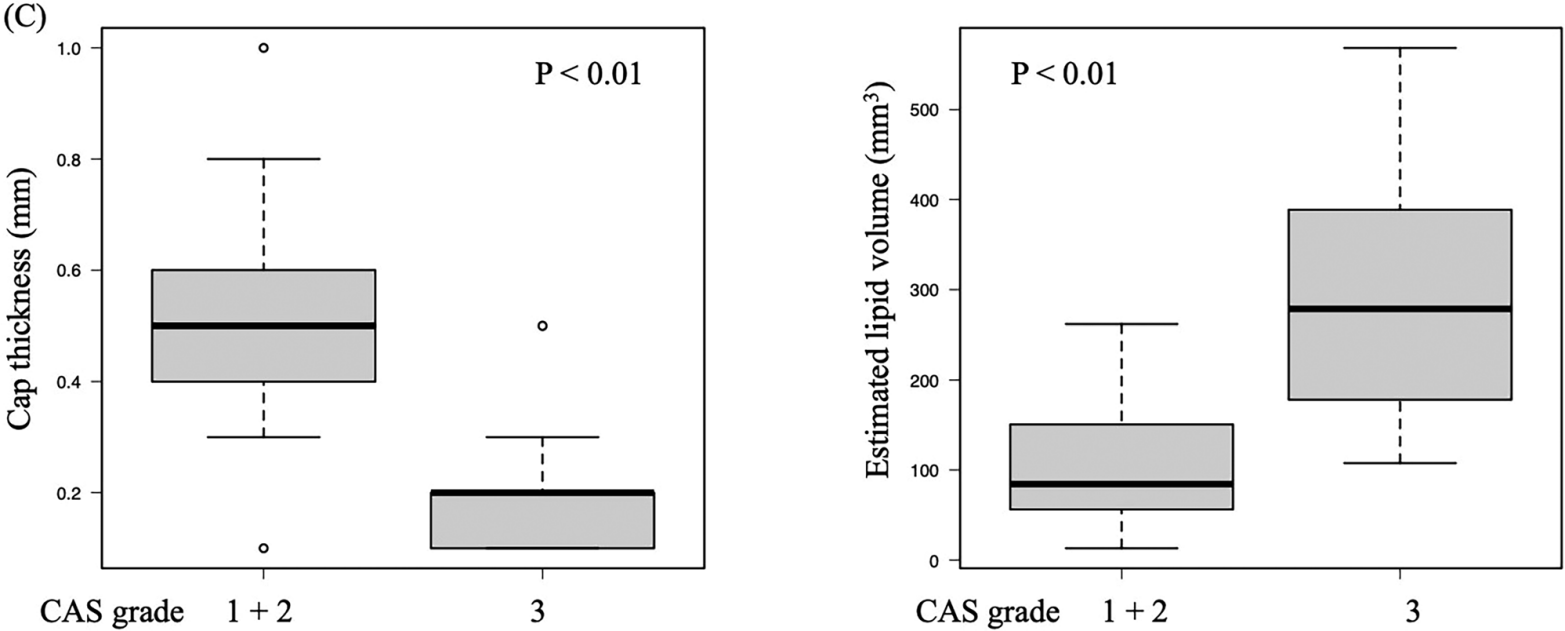
Association between coronary angioscopy (CAS) grades and plaque characteristics measured by integrated backscatter intravascular ultrasound (IB-IVUS). This figure illustrates the relationship between CAS grades and plaque characteristics evaluated by IB-IVUS at the minimum lumen diameter site. The horizontal axis (1, 2, 3) represents CAS grades, where 1 indicates light yellow plaques, 2 indicates yellow plaques, and 3 indicates dark yellow plaques. **A** Proportions of tissue types, including calcification, dense fibrosis, fibrosis, lipid pool (attenuated), and all lipid pools, showing significant differences across CAS grades. **B** Plaque parameters, such as cap thickness, plaque area, lipid pool area, and estimated lipid volume, vary significantly with CAS grades. **C** Additional analysis comparing the combined CAS grades 1 and 2 group with the CAS grade 3 group, performed using the Mann–Whitney U test. Fibrous cap thickness at the minimum lumen diameter site was significantly thinner, and the estimated lipid volume was significantly larger in the CAS grade 3 group compared to the combined CAS grades 1 and 2 group (both P < 0.01). †Kruskal–Wallis test. ††Bonferroni test for multiple comparisons

The Kruskal–Wallis test was used to assess whether IB-IVUS measurement results differed by CAS grade. Significant differences were observed in the proportions of calcification (P = 0.04), dense fibrosis (P < 0.01), fibrosis (P < 0.01), lipid plaque (attenuated) (P < 0.01), and all lipid pools (P < 0.01) at the MLD cross-section. Additionally, significant differences were found in cap thickness (P < 0.01), plaque area (P < 0.01), lipid pool area (P < 0.01), and estimated lipid volume (P < 0.01) at the MLD cross-section. However, no significant differences were observed for the proportion of lipid pool (P = 0.76) at the MLD cross-section or for stent length (P = 0.59). Furthermore, Bonferroni’s multiple comparison tests identified significant differences in dense fibrosis, fibrosis, lipid pool (attenuated), all lipid pool, cap thickness, lipid pool area, and estimated lipid volume between CAS grades 1–2 and CAS grades 1–3 (Fig. 3A and B). Further analysis comparing the combined CAS grades 1 and 2 group with the grade 3 group revealed that the fibrous cap thickness at the MLD site was significantly thinner in the CAS grade 3 group (P < 0.01), whereas the estimated lipid volume was significantly larger in the grade 3 group (P < 0.01) (Fig. 3C).

## Discussion

### Significance of the study

We identified an association between the qualitative assessment of plaque yellowishness using CAS and the quantitative evaluation of plaques at the MLD site measured by IB-IVUS. The findings of this study demonstrated that plaques with higher yellowishness observed via CAS were associated with a larger estimated lipid volume and thinner fibrous cap thickness at the MLD site, as quantified by IB-IVUS. These results suggest that the presence of highly yellow plaques observed through CAS may serve as an indicator of vulnerable plaques within the same coronary artery.

### Utility of detecting lipid plaques

In the present study, no patients were classified into the CAS grade 0 group, suggesting that cases requiring PCI are highly likely to have lipid plaques to some extent. Yellow plaques are highly vulnerable and carry a greater risk of rupture. Patients with yellow plaques in their coronary arteries have been reported to experience a higher incidence of acute myocardial infarction compared with those with white plaques [1]. Furthermore, higher plaque yellowishness observed via CAS has been associated with increased thrombogenicity [5]. Lesions with highly yellow plaques have also been linked to myocardial injury and transient slow flow following coronary stent placement [9]. Vulnerable plaques identified by IB-IVUS are considered predictors of acute coronary syndrome, [10] and the volume of lipid plaques measured by IB-IVUS has been associated with the prediction of no-reflow phenomena after PCI [7]. Thus, the detection of vulnerable lipid plaques holds significant clinical importance when using either CAS or IB-IVUS.

### Methods for detecting lipid-rich plaques

Modalities for detecting lipid-rich plaques include grayscale IVUS, IB-IVUS, near-infrared spectroscopy-IVUS, computed tomography, magnetic resonance imaging, and OCT. In the present study, in addition to CAS, we evaluated the relationship with IB-IVUS, which can be performed simultaneously with grayscale IVUS—commonly used during PCI—and is simple, quick, and convenient to implement during the procedure.

### Consistency and clinical utility of IB-IVUS and CAS findings

In contrast to previous studies on autopsy cases, the present study focused on in vivo observations. Prior research indicated that, in autopsy cases, there was a correlation between plaque yellowishness observed by CAS and fibrous cap thickness measured by IB-IVUS but no association with lipid core size [11]. However, in the present study, plaques with higher yellowishness observed by CAS were associated with larger lipid plaque volumes. This discrepancy may be attributed to differences in the severity of coronary artery lesions evaluated; the previous study primarily examined mild to moderate lesions, whereas our study focused on more severe lesions. Additionally, in our study, we did not evaluate CAS and IB-IVUS findings at precisely the same site, which could have contributed to the differences in results. Nevertheless, in real-world clinical settings of PCI, it is exceedingly difficult to evaluate both modalities at exactly the same location. Therefore, we believe that the methodology used in the present study is more aligned with the practical realities of interventional procedures. The PROSPECT study demonstrated that vulnerable plaques, which are associated with future cardiac events, typically exhibit a combination of smaller minimum lumen area, larger plaque burden, and thin-cap fibroatheroma [12]. Unlike IVUS-based methods, CAS directly visualizes vessel surface characteristics, such as thrombus formation and plaque coloration. Although the plaque yellowishness observed via CAS has traditionally been interpreted as an indicator of fibrous cap thickness, our findings suggest it also correlates with lipid volume within plaques. Thus, CAS may provide a more comprehensive assessment of plaque vulnerability by evaluating plaque yellowishness, which reflects both thin fibrous cap thickness and larger lipid pools. Furthermore, a previous study using CAS (DESNOTE study) demonstrated that patients with higher-grade yellow plaques had worse clinical outcomes, suggesting that plaque yellowishness observed via CAS is clinically relevant and predictive of adverse cardiac events [13]. Compared with the detailed plaque evaluation performed on each cross-section by IB-IVUS, CAS offers the advantage of broader visualization due to the wide field of view provided by the angioscopic catheter and the ability to perform rapid and extensive observations through catheter pullback. This makes CAS particularly valuable for identifying and detecting vulnerable plaques. When suspicious findings indicative of vulnerable plaques are observed, it is feasible to conduct a more detailed evaluation of the specific site using IB-IVUS. We believe that combining these two modalities enhances their effectiveness in detecting and assessing vulnerable plaques. When plaques with high yellowishness are observed via CAS, it may suggest the presence of vulnerable plaques with thin fibrous caps in the same coronary artery. This observation could serve as a prompt to consider aggressive lipid management to mitigate the associated risks. Comprehensive qualitative and quantitative evaluation of intracoronary plaques enables the optimization of procedural strategies in PCI, reducing complication risks and improving long-term outcomes. Furthermore, advancements in these evaluation techniques are expected to facilitate more precise and personalized management of coronary artery disease. Current clinical guidelines recommend that decisions regarding lipid-lowering therapy be primarily based on low-density lipoprotein cholesterol (LDL-C) levels and patient comorbidities. However, the findings of the present study suggest that plaque yellowishness, as assessed by CAS, could serve as a potentially useful marker for identifying vulnerable plaques in clinical practice. CAS findings may help clinicians to more accurately stratify patient risk and justify the rationale for more intensive lipid-lowering therapy, even in patients whose LDL-C levels have already prompted intervention, potentially reducing the risk of future cardiovascular events.

### Limitation

This study had several limitations. First, the sample size was small (n = 36), and the study was a single-center, retrospective observational study. Second, there is a potential for selection bias, as chronic total occlusion lesions and heavily calcified lesions were not included. Additionally, due to the retrospective nature of the study, the cross-sections evaluated by CAS and IB-IVUS did not completely align. Future prospective studies with a larger sample size are warranted to confirm and expand upon our findings.

## Conclusions

Coronary arteries with plaques exhibiting higher yellowishness, as evaluated by CAS, were associated with larger estimated lipid volumes and thinner fibrous caps, as determined by IB-IVUS.

## Data Availability

The datasets generated and/or analyzed during the current study are available from the corresponding author on reasonable request.
